# Phase I and pharmacological study of the farnesyltransferase inhibitor tipifarnib (Zarnestra®, R115777) in combination with gemcitabine and cisplatin in patients with advanced solid tumours

**DOI:** 10.1038/sj.bjc.6602850

**Published:** 2005-10-25

**Authors:** W S Siegel-Lakhai, M Crul, S Zhang, R W Sparidans, D Pluim, A Howes, B Solanki, J H Beijnen, J H M Schellens

**Affiliations:** 1 The Netherlands Cancer Institute/Antoni van Leeuwenhoek Hospital, Plesmanlaan 121, 1066 CX Amsterdam, The Netherlands; 2 Johnson & Johnson Pharmaceutical Research & Development, PO Box 300, Raritan, NJ, USA; 3 Faculty of Pharmaceutical Sciences, Division of Drug Toxicology, Department of Biomedical Analysis, University Utrecht, Sorbonnelaan 16, 3584 CA Utrecht, The Netherlands

**Keywords:** farnesyltransferase inhibitor, phase I study, pharmacokinetics

## Abstract

This phase I trial was designed to determine the safety and maximum tolerated dose (MTD) of tipifarnib in combination with gemcitabine and cisplatin in patients with advanced solid tumours. Furthermore, the pharmacokinetics of each of these agents was evaluated. Patients were treated with tipifarnib b.i.d. on days 1–7 of each 21-day cycle. In addition, gemcitabine was given as a 30-min i.v. infusion on days 1 and 8 and cisplatin as a 3-h i.v. infusion on day 1. An interpatient dose-escalation scheme was used. Pharmacokinetics was determined in plasma and white blood cells. In total, 31 patients were included at five dose levels. Dose-limiting toxicities (DLTs) consisted of thrombocytopenia grade 4, neutropenia grade 4, febrile neutropenia grade 4, electrolyte imbalance grade 3, fatigue grade 3 and decreased hearing grade 2. The MTD was tipifarnib 200 mg b.i.d., gemcitabine 1000 mg m^−2^ and cisplatin 75 mg m^−2^. Eight patients had a confirmed partial response and 12 patients stable disease. No clinically relevant pharmacokinetic interactions were observed. Tipifarnib can be administered safely at 200 mg b.i.d. in combination with gemcitabine 1000 mg m^−2^ and cisplatin 75 mg m^−2^. This combination showed evidence of antitumour activity and warrants further evaluation in a phase II setting.

Tipifarnib (Zarnestra®, R115777; Johnson & Johnson Pharmaceutical Research and Development, Titusville, NJ) is a farnesyltransferase inhibitor (FTI) that can be administered orally. It exerts its antitumour activity by preventing post-translational farnesylation required for activation of selected proteins. Farnesyltransferase inhibitors were initially designed to inhibit the post-translational modification necessary for Ras activation (Kato *et al*, 1992), but their mechanism of action seemed to be more complex involving also other multifunctional proteins (Prendergast *et al*, 1994; Sepp-Lorenzino *et al*, 1995; Maltese, 1998; Du and Prendergast, 1999). Accumulating data have identified three polypeptides whose inhibition may be the basis for the cytotoxic actions of FTIs. These are polypeptides associated with the phosphoinositide 3-OH kinase/AKT pathway (Jiang *et al*, 2000); G protein Rho B, which regulates cytoskeletal organisation (Lebowitz and Prendergast, 1998); and the centromeric polypeptides CENP-E and CENP-F, which interact with microtubules and are necessary for the completion of mitosis (Ashar *et al*, 2000). Tipifarnib has shown good anticancer activity in preclinical *in vitro* and *in vivo* studies (End *et al*, 2001), and has subsequently been evaluated in single agent phase I trials in patients with non-small-cell lung cancer (NSCLC), cervix, colorectal, pancreatic cancer and leukaemia (Zujewski *et al*, 2000; Karp *et al*, 2001; Crul *et al*, 2002). The most common regimen for this agent in solid tumours is 300 mg b.i.d. for 21 consecutive days with 1 week off. Myelosuppression, manifested typically as neutropenia, was the most common toxicity. Other toxicities included fatigue, nausea, vomiting and diarrhoea, which were usually mild in severity. In a continuous schedule, sensory neuropathy was dose limiting. Several phase II trials have demonstrated activity in patients with breast cancer, malignant glioma and acute myelogenous leukaemia (AML) (Cloughesy *et al*, 2002; Johnston *et al*, 2003; Gotlib, 2005). Tipifarnib has also been investigated in two phase III trials in patients with pancreatic cancer and colorectal cancer (Rao *et al*, 2004; Van Custem *et al*, 2004), but no improved efficacy could be demonstrated. Currently, the combination of tipifarnib with several classes of antineoplastic drugs is investigated. Preclinically, the combination of tipifarnib with gemcitabine exhibited synergy (Janssen Research Foundation, 2003), whereas the combination with cisplatin was additive (Skrzat *et al*, 1999). Good activity of combined gemcitabine and cisplatin has been demonstrated in a number of malignancies, including NSCLC (Abratt *et al*, 1997; Crino *et al*, 1997), head and neck cancer (H/N) (Hitt *et al*, 1998), urothelial (Kaufman *et al*, 2000; van der Maase *et al*, 2000) and cervical cancer (Burnett *et al*, 2000). On the basis of the preclinical data and the different mechanisms of antitumour activity of gemcitabine, cisplatin and tipifarnib, a phase I trial was performed of this combination. Since the compounds have some, but potentially important, overlap in toxicity profiles (myelosuppression), a relatively short administration of tipifarnib of 7 days was chosen. The primary objectives were: (i) to determine the maximum-tolerated (MTD) dose, (ii) to characterise the dose-limiting toxicities (DLTs) and (iii) to investigate the pharmacokinetics of each agent, in particular a possible influence of tipifarnib on the pharmacokinetics of gemcitabine and cisplatin.

## PATIENTS AND METHODS

### Eligibility

Patients were eligible if they had a histologically or cytologically confirmed advanced solid tumour for which no curative therapy exists. Other eligibility criteria included a WHO performance status of 0–2 and age ⩾18 years. Previous radiotherapy or anticancer chemotherapy had to be discontinued for ⩾4 weeks before entry into the study, or 6 weeks in case of nitrosourea or mitomycin C. All patients had to have acceptable bone marrow function, defined by neutrophil counts (ANC) ⩾1500 *μ*l^−1^, platelets ⩾100 000 *μ*l^−1^ and Hgb ⩾5.6 mmol l^−1^; and adequate hepatic and renal function defined as creatinine clearance ⩾50 ml min^−1^, total bilirubin ⩽1.5 × upper limit of normal and ASAT and ALAT ⩽2.5 times the normal upper limit (or ⩽5 times the normal upper limit in case of hepatic metastases). The study protocol was approved by the Medical Ethics Committee of the hospital and all patients had to give written informed consent.

### Treatment plan and study design

Tipifarnib was supplied by Johnson & Johnson Pharmaceutical Research and Development as 100 mg tablets. Gemcitabine and cisplatin were supplied by the hospital pharmacy and were used according to local regulatory requirements. Tipifarnib was administered orally twice daily, with intervals of 12 h. Tipifarnib was given on days 1–7 of each 21-day cycle. Gemcitabine was given as a 30-min i.v. infusion on days 1 and 8 and cisplatin was given as a 3-h i.v. infusion on day 1, beginning 30 min after completion of the gemcitabine administration. To minimise nephrotoxicity, a pre-and posthydration schedule was implemented on day 1. Pre- and posthydration consisted of 2000 ml NaCl 0.45%/glucose 2.5% over 14 h before treatment and 3000 ml NaCl 0.45%/glucose 2.5% over 18 h after cisplatin infusion. To determine the influence of tipifarnib on the pharmacokinetics of gemcitabine and cisplatin, administration of this agent was omitted on day 1 of cycle 2. An interpatient dose escalation scheme was used, starting from tipifarnib 100 mg b.i.d., gemcitabine 750 mg m^−2^ and cisplatin 75 mg m^−2^. At least three patients were treated at each dose level. If DLT occurred in a patient during cycle 1, three additional patients were enrolled in that cohort. MTD was defined at which 2 or more out of 6 patients (2⩾6) experienced DLT. Dose-limiting toxicity was defined as drug-related nonhaematological toxicity ⩾grade 3 (excluding untreated nausea and vomiting), grade 4 granulocytopenia lasting >5 days, or associated with fever/infection, grade 4 thrombocytopenia, interruption of tipifarnib dosing for >4 days because of toxicity and grade ⩾2 neurotoxicity or ototoxicity, which did not improve to grade 1 or less within 3 weeks. These toxicities were only considered DLT if they occurred during the first cycle of treatment. In the case of grade 3–4 haematological toxicities, the dose of gemcitabine was adjusted in the next cycle based on nadir blood cell counts. Dose adjustments were relative to the starting dose of gemcitabine received in the previous cycle of therapy. If the nadir granulocyte count was >500 *μ*l^−1^ and the nadir platelet count >50 000 *μ*l^−1^, 100% of the planned gemcitabine dose was administered. If the nadir granulocyte count was ⩽499 *μ*l^−1^ and/or the nadir platelet count ⩽49999 *μ*l^−1^, 75% of the planned gemcitabine dose was administered. The dose of cisplatin was not reduced because of haematological toxicities encountered in the previous cycle. The administration of tipifarnib was discontinued when haematological DLT occurred. After recovery to at least grade 1, tipifarnib was reinstituted at the same dose level in conjunction with a dose modification of gemcitabine. If a patient continued to experience haematological DLT after one dose modification of gemcitabine, the dose of tipifarnib was decreased with 100 mg b.i.d. If at any time the dose of tipifarnib was to be reduced to <100 mg b.i.d. or if retreatment needed to be delayed >2 weeks after the scheduled restarting of a cycle, the patient went off-study. In the case of grade 3–4 nonhaematological toxicities, the dose of both gemcitabine and cisplatin was adjusted. Dose adjustments were relative to the starting dose of gemcitabine and cisplatin received in the previous cycle of therapy. If grade 3 nonhaematological toxicities occurred, patients received 75% of the planned dose. If grade 4 nonhaematological toxicities occurred, patients went off-study. No dose adjustments were made for transaminase elevation unless associated with clinical signs or symptoms. If a grade 3 nonhaematological DLT occurred (or grade 2 neurotoxicity or ototoxicity), treatment with tipifarnib was interrupted until the toxicity resolved to grade 1 and was reinstituted at the discretion of the investigator; a dose modification of gemcitabine/cisplatin as well as a dose reduction of tipifarnib by 100 mg b.i.d. was applied. If at any time the dose of tipifarnib was to be reduced to <100 mg b.i.d. or if retreatment needed to be delayed >2 weeks after the scheduled restarting of a cycle, the patient went off-study.

Patients with progressive disease were excluded from further treatment, and patients who were excluded within the first 21 days for reasons other than drug-related toxicity were replaced.

### Patient evaluation and follow-up

Complete patient history, physical examination, haematology, chemistry, urinalysis and electrocardiogram were performed at baseline and before each cycle of treatment. Physical examination, haematology and chemistry were also evaluated on days 8 and 15 of each cycle. Audiometry was performed at baseline and was repeated if clinically indicated. Indicator lesions were measured before start of treatment and every two cycles, as a basis for the assessment of activity of the treatment. All toxicities observed were graded according to the Common Toxicity Criteria (NCI-CTC, Version 2.0, 1998).

### Pharmacokinetics

Pharmacokinetic studies were performed for each agent during day 1 of the first cycle and for cisplatin and gemcitabine also during day 1 of the second cycle. In the second cycle, tipifarnib was not administered on day 1, to allow for a comparison of gemcitabine and cisplatin pharmacokinetics with and without tipifarnib coadministration. In cycle 2, tipifarnib pharmacokinetics was then determined on day 2. For gemcitabine, 2 ml blood samples were taken at 0, 15, 30, 35, 45, 60, 90 min and at 2, 2.5, 24, 32 and 48 h after the start of the 30 min infusion during cycle 1 and at 0, 15, 30, 35, 45, 60, 90 min during cycle 2. Both gemcitabine and its metabolite 2′, 2′ -difluoro-2′-deoxy-uridine (dFdU) were measured in plasma. Of each blood sample, 1 ml was added to 10 *μ*l of tetrahydrouridine (10 mg ml^−1^), after which it was centrifuged for 5 min at 4°C and 1500 *g*. Subsequently, the plasma layer was stored at −20°C until analysis. Additionally, 15 ml blood samples were taken at 0, 1.5, 4 and 24 h after the start of the gemcitabine infusion for the determination of the triphosphate metabolite of gemcitabine (dFdCTP) in WBC (Sparidans *et al*, 2002). Isolation of WBC was performed using a Ficoll density gradient (Pharmacia, Sweden) as described previously (Heinemann *et al*, 1998). All gemcitabine levels were measured using a validated high-performance liquid chromatography (HPLC) method, analogous to the method of Freeman *et al* (1995). For cisplatin, 5 ml blood samples were obtained at 0, 1.5, 3, 3.25, 3.5, 4, 5, 6.5, 10.5 and 23 h after the start of the 3 h infusion. Blood samples were immediately centrifuged for 5 min at 4°C and 1500 *g*. Unbound platinum was obtained by ultrafiltration using the MPS-1 system equipped with 3 kDa YMT membranes (Amicon Division, Danvers, MA, USA). The resulting plasma ultrafiltrate and total plasma were immediately stored at −20°C until analysis by atomic absorption spectrometry (AAS) (Van Warmerdam *et al*, 1995). At 0, 4 and 23 h after start of the cisplatin infusion, 15 ml blood was collected from which WBC were isolated for the measurement of platinum-DNA adducts by a sensitive and validated ^32^P-postlabelling assay, enabling the selective determination of Pt-GG and Pt-AG adducts (Pluim *et al*, 1999). For tipifarnib, 5 ml blood samples were drawn at 0, 1, 2, 3, 5, 8 and 12 h after the morning dose. Immediately after collection, the blood samples were centrifuged for 5 min at 4°C at 1500 *g*. Separated plasma was stored at −20°C for subsequent drug analysis by a validated HPLC method with UV detection (Zujewski *et al*, 2000).

### Pharmacokinetic analysis

The following pharmacokinetic parameters were determined using noncompartmental analysis with WinNonLin software (version 4.1, Pharsight Corporation, Mountain View, CA, USA): the maximum plasma concentration (*C*_max_), time to maximum plasma concentration (*t*_max_), the elimination half-life (*t*_1/2_), the area under the plasma concentration–time curve from 0 to 48 h (AUC_0–48_) of gemcitabine and dFdU and the AUC_0–23_ for total and unbound (free) platinum. Platinum concentrations obtained by AAS were back-calculated to the corresponding cisplatin concentrations by multiplication with the molecular weight. In addition, the AUC_0–24_ of dFdCTP and the AUA_0–23_ (area under the adduct curve) (Schellens *et al*, 1996) of Pt-DNA adducts were calculated in WBC. For tipifarnib, *C*_max_, *t*_max_ and AUC_0-12_ were determined.

### Statistical analysis

The primary pharmacokinetic parameters of interest for the statistical analyses were *C*_max_ and AUC. Only data from patients who completed the pharmacokinetic blood sampling during the first and second cycles and had no dose reduction were included in the analyses. The *C*_max_ and AUC values of the different drugs were compared between cycle 1 (gemcitabine/cisplatin in the presence of tipifarnib) and cycle 2 (gemcitabine/cisplatin in the absence of tipifarnib) using an ANOVA test (Patnaik *et al*, 2003). A significance level of 0.05 was used for all analyses. The statistical analyses were performed using the SAS statistical software program (version 6.12; SAS Institute, Inc., Cary, NC, USA).

## RESULTS

### Patient characteristics

A total of 31 patients were included at five different dose levels (Table 1): 14 male and 17 female, with a median age of 58 years (range 26–69). Most patients (26 out of 31) had a good performance status of 0–1 (Table 2). A total of 18 patients had prior systemic chemotherapy and two of these patients received previous cisplatin. On the first dose level, one patient was withdrawn from the study before completing the first cycle, due to clinical deterioration, and was replaced. Another patient had received an incorrect dosage and was retrospectively not evaluable for DLT. At the next level, one of the first three patients experienced DLT, and three more patients were entered. In this second cohort, one patient developed severe toxicity (as described under nonhaematological toxicity, transient grade 3 elevation ALAT) during cycle 2 and therefore this was not considered as DLT. For safety reasons, another three patients were included at this level. This was in agreement with the Institutional Review Board (IRB). Two patients included at dose level 2 were replaced because they did not complete the first cycle due to non-drug-related complications. After the evaluation of all patients included at level 2, normal dose escalation was resumed. This decision was supported by results of a concurrent trial of tipifarnib in combination with gemcitabine and cisplatin in a different schedule, which had reached higher dose levels already (Adjei *et al*, 2003b). At dose level 3, three patients were included initially and no DLTs were observed. This was also the situation for the first three patients that were included at dose level 4. At dose level 5, two of the three patients included experienced DLT, and the dose was lowered to the previous level. At dose level 4, three additional patients were treated. One of these patients did not complete cycle 1 and was replaced. Two patients experienced DLT. For safety reasons, it was decided that dose level 3 was the MTD. At this level, gemcitabine was given at 1000 mg m^−2^, cisplatin at 75 mg m^−2^ and tipifarnib at 200 mg b.i.d. Three extra patients were included at the MTD level and one DLT was observed.

### Haematological toxicity

All patients were evaluable for toxicity and 27 patients (87%) experienced grade 3–4 adverse events. A summary of the treatment emergent grade 3–4 haematological and nonhaematological toxicities are shown in Table 3a and the observed DLTs are depicted in Table 3b. The main haematological grade 3 or 4 toxicities were neutropenia (32%, one patient had febrile neutropenia (3%)) and thrombocytopenia (16%). One patient treated at dose level 1 had a grade 3 neutropenia. At dose level 2, several cases of grade 3–4 haematological toxicities were observed, but only thrombocytopenia occurred in cycle 1 and was considered to be a DLT. At dose level 3 (MTD), patients also experienced grade 3–4 haematological toxicities. One patient developed dose-limiting grade 4 thrombocytopenia during cycle 1. At dose level 4, several severe haematological events were observed. Two patients developed a DLT that consisted of neutropenia grade 4 in one patient and febrile neutropenia grade 4, thrombocytopenia grade 4 and electrolyte imbalance grade 3 in the other patient. At dose level 5, patients developed grade 3–4 haematological toxicities but not during cycle 1. Therefore, these events were not considered to be a DLT. Owing to haematological toxicities, three patients (10%) had a dose reduction of tipifarnib and five patients (16%) of gemcitabine.

### Nonhaematological toxicity

The main nonhaematological grade 3 or 4 toxicities were nausea (26%), vomiting (23%) and fatigue (19%). Nausea and vomiting frequently occurred in the first week of each cycle, when tipifarnib was given. In several patients, the intake of tipifarnib was reported as difficult due to this nausea, which appeared worse than that observed in single agent trials (Zujewski *et al*, 2000; Karp *et al*, 2001; Crul *et al*, 2002). At dose-level 1, one patient developed a grade 3–4 pulmonary embolism during cycle 2. At dose levels 2 and 3, several grade 3–4 nonhaematological toxicities were observed, but these events were not considered to be dose limiting as the toxicities developed during subsequent cycles. One patient treated at dose level 2 had a grade 3 elevation of ALAT, but this was transient. This toxicity was not clearly related to the study medication and it was decided to rechallenge the patient at the same doses of the three drugs. This rechallenge proceeded uneventful. At dose level 4, patients experienced also grade 3–4 nonhaematological toxicities. One patient treated at this dose level had a DLT that consisted of neutropenia, thrombocytopenia and electrolyte imbalance. The electrolyte imbalance included grade 3 hypokalemia, hyponatremia and bilirubinemia. It could not be concluded that the electrolyte imbalance was solely induced by the combination administered in this study because this patient received prior cisplatin treatment. The electrolyte imbalance could be induced by previous tubular damage. At dose level 5, patients also developed grade 3–4 nonhaematological toxicities. Two patients experienced DLT consisting of grade 3 fatigue and grade 2 decreased hearing, respectively. Owing to nonhaematological toxicities, one patient (3%) had a dose reduction of tipifarnib and four patients (13%) of gemcitabine and cisplatin.

### Audiometric evaluations

Three patients had a normal value for audiometric examination at baseline, but an abnormal evaluation at a later examination. The first patient was treated at dose level 2 (75 mg m^−2^ cisplatin) and had a grade 2 tinnitus in cycle 6. The other patient was treated at dose level 4 (100 mg m^−2^ cisplatin) and developed a grade 2 tinnitus in cycle 2. For the patient treated at dose level 5 with the grade 2 decreased hearing observed in cycle 1 that was considered to be a DLT, audiometry was not evaluated further.

### Pharmacokinetics

Plasma samples for pharmacokinetic studies were obtained from 30 patients for cisplatin, from 23 patients for Pt-adduct analyses in WBC, from 29 patients for gemcitabine, from 26 patients for gemcitabine in WBC and from 23 patients for tipifarnib. Not all pharmacokinetic parameters could be determined for each patient during cycles 1 and 2 because of an incomplete pharmacokinetic profile or because of a dose reduction. The plasma pharmacokinetic parameters of total and unbound cisplatin following an i.v. infusion of 75 or 100 mg m^−2^ cisplatin with or without tipifarnib are shown in Table 4a. The parameter values of 100 mg m^−2^ were adjusted to 75 mg m^−2^ cisplatin. There were no significant differences in *C*_max_ and AUC_0–23_ of total (*P*=0.124 and *P*=0.575, respectively) and unbound (*P*=0.898 and 0.272, respectively) cisplatin between cycles 1 and 2.

The AUA_0–23_ values of Pt-AG and Pt-GG were also adjusted to the 75 mg m^−2^ cisplatin dose. For Pt-AG, the AUA_0–23_ values were in the ranges of 0.76–10.6 fmol h *μ*g^−1^ DNA and 0.63–13.1 fmol h *μ*g^−1^ DNA with and without tipifarnib, respectively. For Pt-GG, the AUA_0–23_ values were in the ranges of 7.38–34.1 fmol h *μ*g^−1^ DNA and 5.70–34.1 fmol h *μ*g^−1^ DNA with and without tipifarnib, respectively. The AUAs of Pt-AG and Pt-GG were not significantly (*P*=0.769 and *P*=0.715, respectively) affected by the administration of tipifarnib.

The pharmacokinetic parameters of gemcitabine, dFdU and dFdCTP following an i.v. infusion of 750 or 1000 mg m^−2^ gemcitabine with or without tipifarnib are shown in Table 4b. The parameter values of 1000 mg m^−2^ were adjusted to 750 mg m^−2^ gemcitabine. There were no significant differences in *C*_max_ and AUC_0–48_ of gemcitabine (*P*=0.197 and *P*=0.200, respectively) and in AUC_0–24_ of dFdCTP (*P*=0.303) between cycles 1 and 2. However, for dFdU, a significant difference was found in *C*_max_ and AUC_0–48_ (*P*=0.028 and *P*=0.032, respectively) between cycles 1 and 2.

The plasma pharmacokinetic parameters of tipifarnib following a single 200 mg dose of tipifarnib as monotherapy or with gemcitabine/cisplatin are shown in Table 4c. There were no statistically significant differences in *C*_max_ and AUC_0–12_ (*P*=0.094 and *P*=0.918, respectively) between mono- or combination therapy.

### Response

The best response after initiation of therapy is shown in Table 5. Of the 31 patients, 27 patients had at least one postbaseline response evaluation. Eight patients achieved a confirmed partial response. Of these eight patients, two had pancreatic cancer, two had ovarian cancer, two had anal carcinomas, one had oesophagus cancer and one patient had ACUP (adenocarcinoma of unknown primary). The duration of the partial response was at least 8 weeks in five patients. Two patients discontinued treatment after five cycles due to adverse events in one patient and no further clinical benefit in the other patient. Another patient with partial response discontinued after 10 cycles because of no further clinical benefit and two other patients discontinued after six cycles due to the physician decision. In addition to the eight objective responders, 12 patients remained stable for more than 8 weeks. Of these 12 patients, one patient had stable disease for more than 6 months prior to disease progression. Six patients had disease progression and one patient was not evaluable for tumour response.

## DISCUSSION

The FTIs represent a novel class of small molecule inhibitors of cell signalling. Recently, studies have been reported of single agent tipifarnib with negative results (Macdonald *et al*, 2002; Adjei *et al*, 2003a; Rao *et al*, 2004). In this study, tipifarnib in combination with chemotherapy was investigated. The rationale for evaluating the combination of tipifarnib with gemcitabine and cisplatin was the preclinical synergistic cytotoxicity observed between gemcitabine and tipifarnib (Janssen Research Foundation, 2003) and the good clinical combination profile of cisplatin and gemcitabine (Abratt *et al*, 1997; Crino *et al*, 1997; Hitt *et al*, 1998; Burnett *et al*, 2000; Kaufman *et al*, 2000; van der Maase *et al*, 2000). The primary objective of this study was to determine the safety and MTD of twice daily oral dosing of tipifarnib for 7 consecutive days, in combination with i.v. gemcitabine (days 1 and 8) and cisplatin (day 1) of each 21-day cycle. The schedule of gemcitabine followed by cisplatin used in this study was selected because this is the standard schedule used most frequently in the clinic for advanced NSCLC (Soto-Parra *et al*, 2000). Tipifarnib was administered for 7 consecutive days and as monotherapy this regimen was safe up to at least 300 mg b.i.d. in cancer patients (Zujewski *et al*, 2000). However, in view of the overlapping toxicity (myelosuppression) of the drugs, a conservative starting dose of 100 mg b.i.d. was chosen for this trial, thus allowing a substantial safety margin. Previously, a phase I trial of continuous tipifarnib in combination with gemcitabine, given at 1000 mg m^−2^ on days 1, 8 and 15 every 4 weeks, has been performed. In this study, the recommended dose was 200 mg b.i.d. and there was no pharmacokinetic interaction observed (Patnaik *et al*, 2003). In addition, a phase I trial with tipifarnib given for 14 days in combination with gemcitabine on days 1 and 8 and cisplatin on day 1 was also evaluated (Adjei *et al*, 2003b). In this schedule, 300 mg b.i.d. tipifarnib in combination with 1000 mg m^−2^ gemcitabine and 75 mg m^−2^ cisplatin was defined as the MTD. However, it was noted that with repeated administration, the doses of gemcitabine and cisplatin had to be reduced in nine out of 10 patients treated at the MTD because of nausea, vomiting and fatigue. In our trial, the recommended dose is tipifarnib 200 mg b.i.d. for 7 days, in combination with the standard doses of gemcitabine and cisplatin. Clearly, the dose of 300 mg b.i.d. of tipifarnib is too high in combination with the standard doses of cisplatin and gemcitabine on a day 1–7 schedule, as determined in our trial, or on a day 1–14 schedule, as established by others (Patnaik *et al*, 2003). The main DLT was myelosuppression. Dose-limiting nonhaematological toxicities included electrolyte imbalance, fatigue and ototoxicity. The latter was most likely attributable to the fairly high dose of cisplatin (100 mg m^−2^), which was administered at the two highest dose levels. In this study, audiometry was only performed at baseline and if clinically indicated, it is likely that the level of ototoxicity was under-reported. As one patient developed a DLT consisting of grade 2 decreased hearing, it is recommended to formally assess ototoxicity in future studies using this combination. The electrolyte imbalance could be due to previous tubular damage because the patient received prior cisplatin treatment.

No drug–drug interaction between tipifarnib and total and unbound cisplatin was observed in this clinical study. This was as expected because cisplatin is mainly eliminated by the kidneys and has no known enzyme catalysed metabolism.

No clear difference in the DNA-adduct levels in the presence or absence of tipifarnib was found. This was also as expected because the concentrations of cisplatin with or without tipifarnib showed not much difference.

There was no drug–drug interaction between tipifarnib and gemcitabine and dFdCTP. This is consistent with the data from the previous trial (Patnaik *et al*, 2003). However, in our study, a significant difference was found between tipifarnib and dFdU. Gemcitabine is metabolised to its active metabolite dFdCTP by deoxycytidine kinase and can be deactivated to dFdU by deoxycytidine deaminase, whereas tipifarnib undergoes glucuronidation and oxidation by the cytochrome P-450 enzymes. Tipifarnib is predominantly bound to plasma proteins (99%) (Janssen Research Foundation, 2003), whereas the binding of gemcitabine to plasma proteins is negligible (Shipley *et al*, 1992). Therefore, it is unlikely that inhibition of the metabolism of either drug, or their displacement from plasma proteins are potential mechanisms for pharmacokinetic interactions. *In vitro* studies are warranted to unravel the mechanism of interaction between tipifarnib and dFdU. It is expected that the magnitude of the found interaction has limited or no clinical implications.

The pharmacokinetic parameters of tipifarnib were not significantly affected by the concomitant administration of gemcitabine and cisplatin. There was substantial interpatient variability in the pharmacokinetic data of tipifarnib, which has been observed in single agent phase I trials as well (Zujewski *et al*, 2000; Karp *et al*, 2001; Crul *et al*, 2002).

The present trial demonstrated that tipifarnib in combination with gemcitabine and cisplatin is safe and that major and clinically relevant drug–drug interactions were not evident. Consistent with this finding, the current regimen revealed signs of activity in a wide variety of tumours. There were eight confirmed partial responses and 12 patients remained stable for more than 8 weeks. As this study represents a combination of tipifarnib with an effective cytotoxic regimen, the promising efficacy results documented in this study also have to be interpreted with caution. Nonetheless, phase II studies of this combination in a number of solid tumours are warranted. It is of interest to determine if this combination has equal or greater effect than the standard treatment of gemcitabine and cisplatin alone and more information is needed about the mechanism of action of tipifarnib to select potential surrogate markers to determine if the recommended dose is also the effective dose.

## Figures and Tables

**Table 1:** Doses of gemcitabine, cisplatin and tipifarnib and number of patients included per dose level

**Dose level**	**Gemcitabine (mg m^−2^) days 1 and 8**	**Cisplatin (mg m^−2^) day 1**	**Tipifarnib (mg twice daily) days 1–7**	***n***
1	750	75	100	4
2	750	75	200	11
3	1000	75	200	6
4	1000	100	200	7
5	1000	100	300	3

**Table 2:** Patient characteristics

	***n***
*Gender*	
Male	14
Female	17

*Age*	
Median (years)	58
Range (years)	26–69

*Race*	
White	30
Black	1

*No. of cycles of tipifarnib+gemcitabine and cisplatin*	
1–2	13
3–4	5
5–6	8
7–8	3
>8	2

*Tumour types*	
Pancreatic	9
ACUP (adenocarcinoma of unknown primary)	6
Ovary	4
Colorectal	3
Gastric	2
Anal canal	2
Bile duct carcinoma	1
Head and neck	1
Mesothelioma	1
Sarcoma	1
Oesophagus	1

*Performance status*	
0	10
1	16
2	5

*Previous therapy*	
Surgery	19
Radiotherapy	8
Chemotherapy	18

**Table 3a:** Incidence of treatment emergent Grade 3 or 4 haematological and nonhaematological toxicities for all cycles observed

	**Level 1**	**Level 2**	**Level 3**	**Level 4**	**Level 5**	**Total**
Cohort	1	2	3	4	5	
	*n*	*n*	*n*	*n*	*n*	*n* (%)

Total no. of subjects	4	11	6	7	3	31
No. of subjects with Grade 3–4 toxicity	2	11	4	7	3	27 (87)

*Haematological*						
Neutropenia^a^	1	3	2	4	1	11 (35)
Anaemia	—	3	—	1	—	4 (13)
Thrombocytopenia	—	1	2	1	1	5 (16)

*Nonhaematological*						
Nausea	—	3	2	2	1	8 (26)
Vomiting	—	3	2	2	—	7 (23)
Constipation	—	—	—	2	—	2 (6)
Fatigue	—	2	1	1	2	6 (19)
Hypokalemia	—	—	—	3	—	3 (10)
Thrombophlebitis	—	2	—	—	—	2 (6)
Pulmonary embolism	1	—	—	—	—	1 (3)
ALAT elevation	—	1	—	—	—	1 (3)

a One patient treated at dose level 4 developed febrile neutropenia.

**Table 3b:** Dose-limiting toxicities in cycle 1

	**Level 1**	**Level 2**	**Level 3**	**Level 4**	**Level 5**	**Total**
Cohort	1	2	3	4	5	*n*

No. of subjects evaluable for DLT	2/4	9/11	6/6	6/7	3/3	26/31
Subjects with DLT	—	1	1	2	2	6

*Haematological*						
Neutropenia	—	—	—	1	—	1
Febrile neutropenia	—	—	—	1	—	1
Thrombocytopenia	—	1	1	1	—	3

*Nonhaematological*						
Bilirubinemia	—	—	—	1	—	1
Fatigue	—	—	—	—	1	1
Ototoxicity	—	—	—	—	1	1
Hypokalemia	—	—	—	1	—	1
Hyponatremia	—	—	—	1	—	1

**Table 4a:** Mean (s.d.) pharmacokinetic parameters of total and unbound cisplatin, with or without tipifarnib (100–300 mg)

**Variable**	**Units**	**Gem/cis (s.d.) *n*=24**	**gem/cis+tipifarnib (s.d.) *n*=30**	***P*-value^a^**
*Total cisplatin*				
Median *t*_max_	Hours	3.19	3.28	–
*C*_max_^b^	*μ*g ml^−1^	4.72 (1.68)	4.29 (0.78)	0.124^c^
AUC_0-23_^b^	h *μ*g ml^−1^	63.0 (8.93)	61.6 (9.78)^d^	0.575^c^
*t*_1/2_	Hours	40.6 (13.2)^e^	46.1 (18.4)^c^	—

*Unbound cisplatin*				
Median *t*_max_	Hours	3.03	3.02	—
*C*_max_^b^	*μ*g ml^−1^	1.91 (1.77)	1.59 (0.24)	0.898^c^
AUC_0-23_^b^	h *μ*g ml^−1^	6.84 (3.06)	5.80 (1.93)^f^	0.272^g^
*t*_1/2_	Hours	9.01 (8.78)^h^	6.65 (18.2)^i^	—

*Pt-AG*				
AUA_0-23_^b^	fmol h *μ*g^−1^	2.78^j^	2.66^g^	0.769^k^

*Pt-GG*				
AUA_0-23_^b^	fmol h *μ*g^−1^	16.70^g^	18.52^g^	0.715^k^

a Two-sided *P*-value for testing a difference between the two treatments. Only data from patients who completed the study were included.

b Parameter values adjusted to 75 mg m^−2^ dose of cisplatin.

c *n*=24.

d *n*=29.

e *n*=17.

f *n*=28.

g *n*=23.

h *n*=12.

i *n*=16.

j *n*=22.

k *n*=15.

Gem=gemcitabine; cis=cisplatin.

**Table 4b:** Mean (s.d.) pharmacokinetic parameters of gemcitabine, dFdU and dFdCTP with or without tipifarnib (100–300 mg)

**Variable**	**Units**	**Gem/cis (s.d.) *n*=23**	**Gem/cis+tipifarnib (s.d.) *n*=29**	***P*-value^a^**
*Plasma gemcitabine*				
Median *t*_max_	Hours	0.50	0.50	—
*C*_max_^b^	*μ*g ml^−1^	12.5 (3.53)	12.6 (2.31)	0.197^c^
AUC_0–48_^b^	h *μ*g ml^−1^	6.71 (2.12)^d^	6.67 (1.07)^e^	0.200^f^
*t*_1/2_	Hours	0.18 (0.06)^d^	0.55 (1.77)^g^	—

*Plasma dFdU*				
Median *t*_max_	Hours	0.6^g^	0.61^h^	—
*C*_max_^b^	*μ*g ml^−1^	25.6 (5.47)^g^	27.0 (4.89)^h^	0.028^g^
AUC_0–48_^b^	h *μ*g ml^−1^	237 (89.4)^d^	274 (119)^e^	0.032^f^
*t*_1/2_	Hours	32.4 (26.2)^g^	10.2 (6.37)^h^	—

*Mononuclear blood cell dFdCTP*				
AUC_0–24_^b^	h nanomol mg protein^−1^	18.3 (40.9)^i^	14.7 (22.9)^j^	0.303^d^

a Two-sided *P*-value for testing a difference between the two treatments. Only data from patients who completed the study were included.

b Parameter values adjusted to 750 mg m^−2^ dose of gemcitabine.

c *n*=22.

d *n*=19.

e *n*=20.

f *n*=14.

g *n*=24.

h *n*=30.

i *n*=21.

j *n*=26.

Gem=gemcitabine; cis=cisplatin.

**Table 4c:** Mean (s.d.) pharmacokinetic parameters of tipifarnib (200 mg) as monotherapy, or with gemcitabine (750–1000 mg m^−2^) and cisplatin (75–100 mg m^−2^)

**Variable**	**Units**	**Tipifarnib (s.d.) *n*=20**	**Tipifarnib+gem/cis (s.d.) *n*=23**	***P*-value^a^**
*Tipifarnib*				
Median *t*_max_	Hours	3.0	2.1	—
*C*_max_	ng ml^−1^	499 (275)	635 (366)	0.094^b^
AUC_0-12_	h ng ml^−1^	2630 (1134)	2971 (2089)^c^	0.918^b^

a Two-sided *P*-value in log-scale for testing a difference between the two treatments. Only data from patients who completed the study were included.

b *n*=20.

c *n*=22.

Gem=gemcitabine; cis=cisplatin.

**Table 5:** Best response during treatment

**Overall response^a^**	**No. of patients**	**% of patients (*N*=27)**
Partial response (PR)	8	30
Stable disease (SD)	12	44
Progressive disease (PD)	6	22
Not evaluable (NE)	1	4

a PR; at least 30% decrease in the sum of the longest diameter of target lesions taking as reference the baseline sum longest diameter.

SD; neither sufficient shrinkage to qualify for PR nor sufficient increase to qualify for PD taking as reference the smallest sum longest diameter since the treatment started.

PD; at least a 20% increase in the sum of the longest diameter of target lesions taking as references the smallest sum longest diameter recorded since the treatment started or the appearance of new lesions.
